# Developing children’s nursing care outcome statements in Africa using World Café methods

**DOI:** 10.1111/inr.12621

**Published:** 2020-09-07

**Authors:** Minette Coetzee, Angela Leonard, Candice Bonaconsa, Nina Power, Natasha North

**Affiliations:** ^1^ Department of Paediatrics and Child Health Faculty of Health Sciences University of Cape Town Cape Town South Africa

**Keywords:** Africa, capacity building, Healthcare Quality Indicators, nursing, paediatric nursing, nursing care, nursing metrics

## Abstract

Nursing metrics use indicators to make the outcomes of nursing care visible through measurement. Metrics must be sensitive to the context‐specific nature of nursing and should reflect the work that nurses really do. A workshop at the Building Children’s Nursing conference (2019) was convened to develop statements of nursing care outcomes and actions specific to the work of children’s nurses in African care settings, using the World Café method and the Nightingale Metrics approach. The process resulted in the development of statements as intended. Appropriate nursing metrics can guide data‐driven practice improvements and decision‐making about supporting the nursing workforce.

## Introduction

Development of nursing metrics to understand and improve care quality is urgently required but is challenging in lower‐resourced, high‐need African settings. Developing appropriate nursing metrics requires consideration of the contexts in which nursing is embedded (Maben et al. 2012).

The biennial International Building Children’s Nursing conference brings together educators, researchers and practice leaders involved in children’s nursing education and workforce development in Africa. The fourth Building Children’s Nursing conference met in Cape Town between 16th–18th April 2019 and was attended by 115 delegates from ten African countries (Botswana, Ethiopia, Ghana, Kenya, Malawi, Namibia, South Africa, Rwanda, Zambia and Zimbabwe) as well as the Netherlands, Britain and America. The conference included a participatory workshop for the purpose of harnessing the insights and expertise of attendees to develop statements of nursing care outcomes and nursing action statements related to the work of children’s nurses in Africa.

## Background

Nursing metrics use indicators to capture the effects of care through measurement (Maben et al. 2012). The drive to develop nursing metrics to understand and improve the quality of care has led to the establishment of increasingly sophisticated measurement systems in higher‐resourced health systems (Maben et al. 2012). Innovations including clinical dashboards in the National Health Service in England and the National Database of Nursing Quality Metrics by the American Nurses Association in the United States of America have enabled the monitoring of outcomes for which nurses can be held accountable.

The Lancet Global Commission on High Quality Health Systems (Kruk et al. 2017) called for the application of science‐led measurable indicators to improve the quality of care in low‐ and middle‐income countries. Governments and global funders investing in children’s nursing workforce development are also keenly interested in understanding the value created in terms of improved care outcomes.

Two substantial reviews of the state of nursing metrics (Dubois et al. 2013; Maben et al. 2012) informed the researchers and enabled specification of three criteria. Contextually appropriate children’s nursing metrics should:

- focus on the work that children’s nurses really do
- capture the difference that children’s nursing care makes to improved health outcomes (distinct from other influences or inputs)
- be in a form that allows the contribution of nurses to children’s care and wider strategic goals to be reported.

The research team identified that a fundamental challenge to developing and implementing children’s nursing metrics in our African settings is to understand the context and actual work of nurses practicing in these contexts. Although children make up between 25 and 45 per cent of the population in many African countries, children’s nurses (registered nurses with an additional specialist qualification in paediatrics) form barely 1% of the nursing workforce. Children needing in‐patient care face many barriers to accessing care and are often treated in adult wards by generalists. Availability of specialist paediatric care is mainly limited to large district and central hospitals located in major urban areas which treat the sickest children. It is usual for children admitted to hospital to be accompanied by a mother or guardian who takes an active role in their care. Children’s nurses’ roles vary between institutions, and children’s nurses often oversee care given by less specialized nursing cadres.

## Applying Nightingale Metrics: What do children’s nurses do?

Florence Nightingale’s 1860 assertion that ‘What nursing has to do … is to put the patient in the best position for nature to act’ continues to provide a foundation for evidence‐based quality measurement. The research team were already aware of the work of Martha Curley and colleagues at Boston Children’s Hospital, which took inspiration from Nightingale’s legacy to develop a contemporary approach to defining the nursing care processes that contribute to optimal patient care outcomes – termed Nightingale Metrics (Curley & Hickey 2006).

**Fig. 1:**
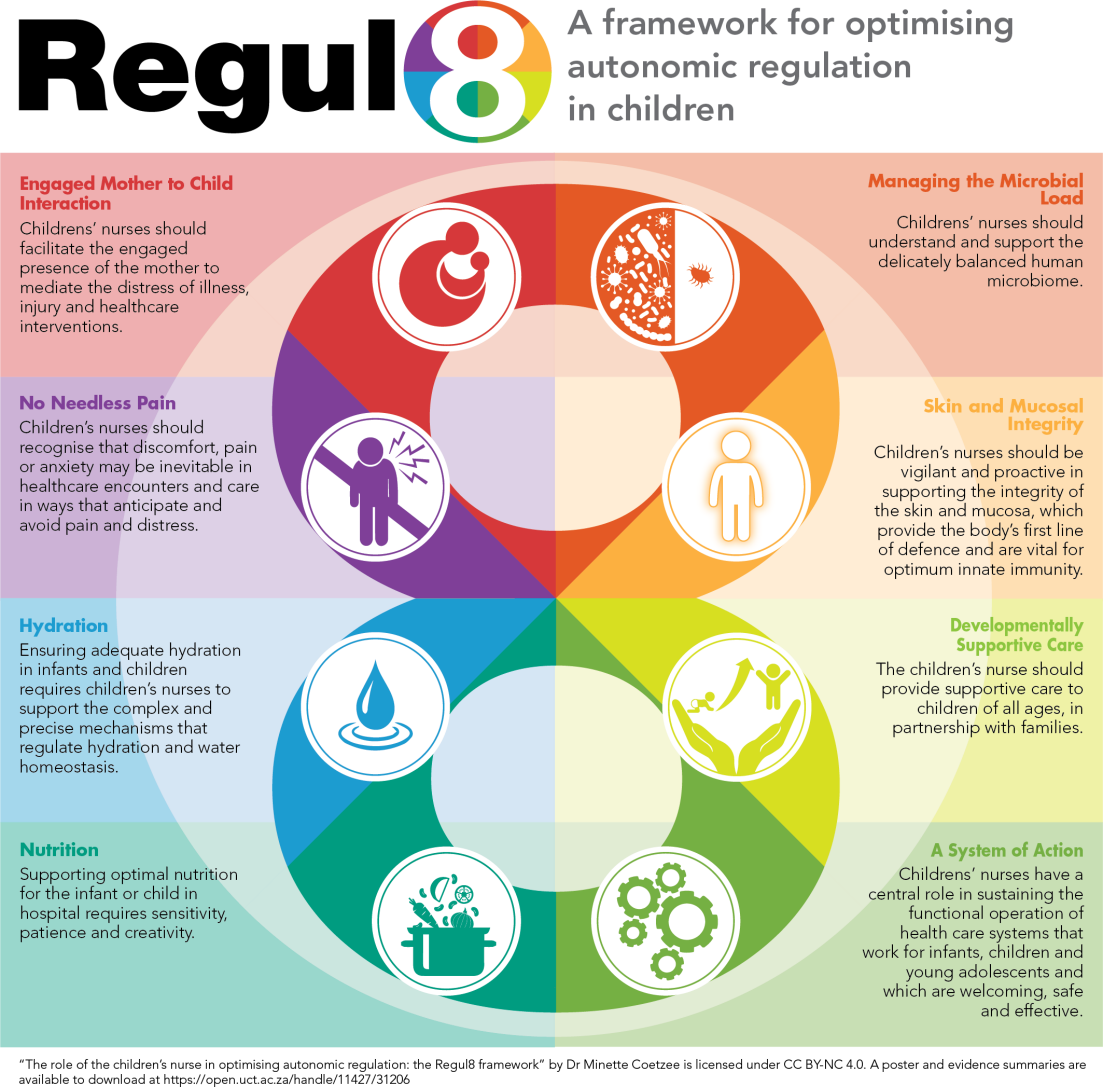
The Eight Domains of the Regul8 Framework (abridged) (Coetzee 2019).

In emphasizing the importance of the nurses’ role in regulating the effects of external factors such as ventilation, hygiene and rest, Nightingale’s theories provided the basis of modern nursing practice. The continued relevance of these theories is underscored by new understandings in the fields of innate immunology, developmental neurobiology and autonomic regulation. Placing hospitalized children in the ‘best position for nature to act’ requires anticipatory, preventative and supportive nursing activities that support and restore self‐regulation of the biological and physiological processes crucial to health and development. This understanding informed the development of the Regul8 framework of nursing care (Coetzee 2019) which provides the basis for curricula in the majority of children’s nursing educational programmes in southern and east Africa (see Fig. 1).

The researchers took the eight outcome domains of the Regul8 framework as a starting point and specified a care outcome statement for each outcome domain (see Table 1). By asking the question ‘What do nurses do to … [achieve the outcome statement]’ the intention was to elicit examples of nursing actions that could provide a basis for the development of contextually appropriate nursing‐sensitive outcome indicators.

**Table 1:** Outcome domains, care outcome statements and children’s nursing metrics statements for children’s nursing in Africa developed at the fourth Building Children’s Nursing for Africa conference, Cape Town, April 2019

Outcome domain	Care outcome statement	Nursing actions (metrics statements)
*Placing the patient and family in the best position for nature to act on them*		*What do children’s nurses do to achieve this outcome?*
Engaged Mother to Child Interaction	A mother and child who are connected	Nurses facilitate mothers’* active participation in caring for the child while mediating wider social and environmental influences affecting the child’s health and wellbeing.
No needless pain	A child who is comfortable and free from pain	Nurses approach each child’s pain on an individual basis, involving the mother in assessing, planning, implementing and evaluating appropriate pharmacological and non‐pharmacological pain management within an inter‐professional team approach. This includes the use of appropriate pain scales and other ways to promote and provide comfort, including through good positioning and sensible clustering of care.
Hydration	A child who has adequate fluid in their body	Nurses support the mother and child in maintaining adequate hydration by encouraging adequate intake of appropriate fluids (including breastmilk) by the most appropriate means, and by monitoring output. Nurses’ actions are guided by the age and weight of the child, within their context and specific need.
Nutrition	A child who is well nourished	Nurses support the mother and child to optimize the child’s nutrition through accurate assessment, age‐appropriate and ability‐specific feeding provision and practices. Nurses enable mothers to address barriers to good nutrition, recognizing the impact of attachment, psycho‐social factors and illness.
Skin and Mucosal Integrity	A child with skin and mucosa that are fully intact	Nurses work with mothers to assess and monitor the integrity of the child’s skin and mucosa on an ongoing basis with a focus on preventing skin and mucosal breakdown. Nurses involve mothers in identifying risks for skin and mucosal breakdown and providing holistic management of problems related to nutrition, hydration, pressure and cleansing agents, providing the necessary care guided by best practice.
Management of Microbial Load	Maintenance of a beneficial microbiome and prevention and treatment of pathogens	Nurses follow best practice guidelines of infection control and the discretionary use of antibiotics and treatment, supporting mothers to achieve healthy outcomes. Nurses actively facilitate immediate skin to skin contact for babies and their mothers, with initiation of breastfeeding as soon as possible to colonize the newborn with beneficial microbiota.
Developmentally Supportive Care	A child who is positioned and stimulated in a way that is right for their current stage of development and anticipates the next stage	Nurses track development in utero and monitor age‐appropriate growth and development before and after birth. Nurses support and educate mothers to facilitate the child’s development by providing information regarding age‐appropriate nutrition and stimulation in order to meet the child’s milestones.
A System of Action	A unit that works	Nurses lead and are active members of inter‐professional teams with a clear, shared vision of best practice, operational and strategic objectives with the child and mother at the centre.

* In our work (North, Leonard, Bonaconsa, Duma, Coetzee, 2020) we have observed that the majority of children in hospital are accompanied by a female relative widely referred to as the child’s mother. Mother is therefore used to refer to any woman accompanying and caring for a child in hospital, whether or not they are the child’s biological mother or a grandmother, aunt, older sister or foster mother.

## Processes to develop nursing care outcome statements

The care outcome statements were presented at a World Café workshop at the Building Children’s Nursing conference. The World Café method facilitates meaningful collaborative dialogue between large groups of stakeholders (The World Café Community Foundation 2019) and has been used successfully to capture insights and expertize from groups of health professionals attending conferences and meetings of professional societies (Cosby et al. 2015; Maskrey & Underhill 2014). Our expectation was that in‐person engagement would be preferred to a survey consultation (such as the Delphi technique) by this group of participants, resulting in increased and better‐quality participation. The process involves seven integrated design principles (The World Café Community Foundation 2019):

- providing participants with a clear context and purpose for the meeting
- creating a safe, hospitable space to stimulate creative thinking and exchange
- exploring ‘questions that matter’
- encouraging contributions through facilitation
- connecting diverse perspectives through deliberate movement between tables and groups
- listening for emerging patterns and insights
- sharing collective discoveries through a ‘harvest’ of key points, eventually resulting in consensus among a smaller group of organizers

Attendees were divided into seven groups according to their paediatric nursing sub‐specialization (medical/general paediatrics; emergency and trauma; paediatric intensive care; long term specialist care; surgical; neonatal intensive care and newborn high care; rehabilitation, primary and community health care). After a familiarization exercise, groups were assigned to one of eight tables for their conversations to begin.

Each table was set up to explore one of eight care outcome domains. Two people (a facilitator and a summariser) were permanently stationed at each table to maintain continuity while stimulating interaction within rotating groups. The facilitator kept the conversation on task and on time. The summariser recorded what each group said, supporting them in reaching consensus as they formulated nursing action statements. Facilitators introduced the care outcome statement to the attendees, asking them to consider what nurses do to achieve the outcome from their perspective as, for example, paediatric intensive care nurses. The groups moved from table to table, and the outcome statements (and the facilitators and summarisers) stayed at the same tables throughout. This movement is a key element of the World Café approach and it ensured that all participants engaged with every statement under development. Twenty minutes was allocated to consider nursing actions in relation to each care outcome domain, with additional time for refining summary statements and presenting and discussing the statements.

## Refining the nursing metric statements

To allow time for collation of the statements, consultation and adoption of the final statements were carried out through a digital consultation after the workshop. The statements generated by participants were reviewed by the research team immediately following the workshop. It was agreed that overall the statements represented an accurate summary of the contributions made by participants. Minor editing was carried out to achieve grammatical consistency and support understanding. Statements that were worded as ‘Nurses carry out…’ etc. and which directly described a nursing action were most effective at conveying the information, and the format of action‐oriented statements starting with ‘Nurses…’ was adopted throughout, with minor re‐wording.

Consensus on the final statements was achieved through a digital consultation of all attendees using Google Forms, a free online survey platform known to be accessible to participants. All comments were reviewed by the research team. Five sets of comments proposing editorial changes to the statements, defined as improving the clarity and consistency of the statements through minor changes to wording, were incorporated. Two sets of comments (provided by MN and BC) proposed substantive edits, defined as representing an important change to the wording which considerably improved the meaningfulness of the statement, and these were accepted. The final statements are presented in Table 1.

## Strengths and limitations

The limitations of this approach lie in the non‐generalizability of the findings. They relate only to the participants in the World Café event and are not the product of an experimental design. However, the concentration of expert children’s nurses at the workshop afforded inclusive participation from this small international community of practitioners. Our assumption that the in‐person engagement offered by the World Café method would be welcomed by this group of participants seemed accurate, as the optional workshop secured participation from over 90% of the 115 conference delegates. While we have reported participants’ contributions as transparently as possible, the resulting statements are framed around the initial domain statements provided by the researchers.

## Conclusion

Application of the Nightingale metrics process provided an effective starting point to establish a foundation for further work that will enable development of metrics and implementation of measurement. The application of the Regul8 framework to structure the outcome domains and the resulting statements generated by attendees embodied a distinctively Afrocentric philosophy of nursing, anticipating substantial engagement and responsibility among family care‐givers. This nonetheless aligned closely with the philosophy of the Nightingale metrics approach, with its emphasis on self‐care and putting patients in the best position for nature to act.

The importance of ensuring that nurses and nursing research provide the basis for developing contextually appropriate nursing metrics has been stated (Dubois et al. 2013; Foulkes 2011). Through this process, we have successfully responded to the fundamental challenge of contextualizing and describing the work that children’s nurses really do in African settings. The product of this process is not yet an implementable metrics framework. The next stage of this process will involve researchers working with children’s nurses in four new Best Practice Units in southern Africa to explore the practicalities of collecting and using information about the practices described in the statements to understand nursing performance.

## Implications for nursing policy and practice

Appropriately developed and implemented nursing metrics can empower nurses to understand and improve their practice and demonstrate the value of nursing through data about care outcomes. In the context of renewed calls to expand the nursing workforce and accelerate specialist nurse training to meet global health needs, nursing metrics can assist nurses, nurse leaders, policymakers and administrators to participate in data‐driven decision‐making to support and develop the nursing workforce.

## Author Contributions

MC was the lead facilitator for the World Café workshop, with design assistance from CB. Additional assistance at the workshop was provided by NN, AL, CB and NP. NN, AL and NP undertook the process of consulting participants about the statements after the workshop, collated comments and revised statements. MC is the originator of the Regul8 framework for children’s nursing care that formed the basis for the statement domains. NN wrote the first draft of this paper, which was then revised by MC. All authors agreed on the final version of the paper.

## Data Availability

A report of the proceedings of the Building Children’s Nursing for Africa conference 2019 can be found at: http://www.childnursingpractice.uct.ac.za/sites/default/files/image_tool/images/198/PDF/ConstructsofCareBCN2019DailyReportsforCNPDIwebsite.pdf.
